# Effects of Haptic Feedback Interventions in Post-Stroke Gait and Balance Disorders: A Systematic Review and Meta-Analysis

**DOI:** 10.3390/jpm14090974

**Published:** 2024-09-14

**Authors:** Maria Gomez-Risquet, Rocío Cáceres-Matos, Eleonora Magni, Carlos Luque-Moreno

**Affiliations:** 1 Facultad de Enfermería, Fisioterapia y Podología, Universidad de Sevilla, 41009 Sevilla, Spain; margomris@alum.us.es (M.G.-R.); rcaceres3@us.es (R.C.-M.); 2 CTS-1137 “Neurological Physiotherapy, Innovative Neurorehabilitation & Neurodevelopment Disorders (NEUROPhysiUS)” Research Group, Universidad de Sevilla, 41009 Seville, Spain; 3 Instituto de Biomedicina de Sevilla (IBiS), Departamento de Enfermería, Universidad de Sevilla, 41009 Seville, Spain; 4 CTS-1050 “Complex Care, Chronicity and Health Outcomes” Research Group, Universidad de Sevilla, 41009 Seville, Spain; 5 CTS-969 “Care Innovation and Health Determinants” Research Group, Universidad de Sevilla, 41009 Seville, Spain; 6 Instituto de Biomedicina de Sevilla (IBiS), Departamento de Fisioterapia, Universidad de Sevilla, 41009 Seville, Spain

**Keywords:** stroke rehabilitation, postural balance, gait disorders, neurologic, feedback, haptic technology

## Abstract

**Background**: Haptic feedback is an established method to provide sensory information (tactile or kinesthetic) about the performance of an activity that an individual can not consciously detect. After a stroke, hemiparesis usually leads to gait and balance disorders, where haptic feedback can be a promising approach to promote recovery. The aim of the present study is to understand its potential effects on gait and balance impairments, both after interventions and in terms of immediate effects. **Methods**: This research was carried out using the following scientific databases: Embase, Scopus, Web of Science, and Medline/PubMed from inception to May 2024. The Checklist for Measuring quality, PEDro scale, and the Cochrane collaboration tool were used to assess the methodological quality and risk of bias of the studies. **Results**: Thirteen articles were chosen for qualitative analysis, with four providing data for the meta-analysis. The findings did not yield definitive evidence on the effectiveness of haptic feedback for treating balance and gait disorders following a stroke. **Conclusions**: Further research is necessary in order to determine the effectiveness of haptic feedback mechanisms, with larger sample sizes and more robust methodologies. Longer interventions and pre–post design in gait training with haptic feedback are necessary.

## 1. Introduction

Motor sequelae are among the most prevalent conditions following a stroke and are the main cause of disability in these patients [1,2]. Mobility problems exist in 70–80% of post-stroke patients, with gait disorders impacting 80% of them [3] and often persisting even after the rehabilitation process [4]. In patients with post-stroke hemiparesis, gait is usually developed with wrong weight adjustment and a decrease in functionality and balance [5]. In fact, balance disorders are another main problem after cerebrovascular accidents, with evidence showing a prevalence of 48.1% among post-stroke patients [6]. These balance impairments often play a significant role in reducing walking ability and increasing the risk of falls in post-stroke patients in both the acute and chronic phases [7]. Specifically, deficits in weight shifting and stepping responses in the mediolateral direction are thought to be linked to more falls in older adults [8]. In line with that, evidence suggests that difficulties with lateral weight shifting and maintaining stability can limit comfortable walking speeds and hinder the ability of chronic stroke survivors to walk faster [5].

There is a growing body of research on the connection and interaction between cognitive and motor functions in motor and balance interventions [9,10]. Innovative paradigms based on the top-down theory have been created in neurorehabilitation to promote cognitive function activation, enhancing the recovery process and integrating multisensory information with augmented feedback [11]. Thus, the main goal of feedback approaches is to provide information about the execution of an activity that an individual cannot consciously perceive [12]. Specifically, evidence highlights the application of feedback mechanisms in both static and dynamic balance interventions [13] as well as gait interventions [14]. Feedback information can be delivered through different types of stimuli, such as visual, auditory, or tactile, among others [15,16]. Even though both visual and auditory feedback are widely used in clinical approaches, evidence has pointed out that potential inhibition or overload can be produced by using these two sensory channels [17]. Haptic feedback stands out as a different mechanism for sharing information in these feedback processes, providing information through the sense of touch [18].

Haptic feedback is an established method for providing sensory information to patients with sensorimotor disorders [19,20,21] and can be classified as tactile cues or kinesthetic information [22]. The former refers to sensations such as vibration [23], pressure, texture [24], or electrical [25], while the latter refers to spatial references provided to the user [26]. Specifically, in the physiotherapy field, haptic feedback is used with the objective of providing precise tactile stimuli to facilitate functional and physical recovery [27], widely in upper limb interventions [28,29]. In fact, some authors have highlighted that haptics can help minimize trunk compensation during upper limb rehabilitation by providing feedback that does not rely on visual cues, verbal instructions, or physical constraints [30].

In other clinical procedures widely used in physiotherapy, such as virtual reality (VR), haptics is also usually implemented [31]. The application of haptics in VR is focused on enhancing the sense of touch and obtaining somatosensory information, allowing the patient a sensation of touch and also giving reinforced feedback [32]. Being part of a larger display, haptics in VR would only constitute an element of a global environment. Thus, the present review focuses on amplifying the knowledge of implementing isolated haptic feedback mechanisms in balance and gait recovery approaches, considering the current interest in feedback mechanisms as an independent entity [33]. This will allow the study of haptic feedback mechanisms without mixing them with other types of feedback that might contaminate the derived conclusions. This is in line with the current evidence, highlighting haptic feedback mechanisms as useful tools for the recovery of motor skills after a stroke [11,34].

The effects of real-time feedback during intervention periods (pre–post designs) and immediate applications (simultaneous application of feedback while measuring study variables) in balance and gait have already been studied in elderly populations [13]. Thus, the primary objective of this study is to explore the different uses and effectiveness of haptic feedback mechanisms in balance and gait disorders in post-stroke patients, inquiring into the following: (1) designs with prolonged interventions over time and pre–post-training measures and (2) designs that provide immediate/simultaneous effects through single-measure assessments. This way, we aim to increase knowledge of haptic feedback in the recovery of two variables that are crucial for post-stroke rehabilitation, as well as to explore new and interesting aspects of haptic feedback effects that could guide future Randomized Controlled Trials (RTCs).

## 2. Materials and Methods

In the present systematic review, the Preferred Reporting Items for Systematic Review and Meta-Analysis (PRISMA) [35] guideline was followed. The complete PRISMA checklist is presented in the Supplementary Material of this article (Table S1). This systematic review was registered in the PROSPERO database (CRD42024557127), where it can be consulted and where the updated versions are available.

### 2.1. Research Strategy

Potentially eligible trials up to May 2024 were reviewed in exhaustive research conducted by two independent reviewers (M.G.-R. and E.M.), and an extra reviewer (C.L.-M.) was considered for consensus when needed. The search of the studies was performed in the following four databases: Medline/PubMed, Embase, Web of Science, and Scopus (search strategies are listed in Table 1). The research strategy included all available records in English and Spanish, and the results were filtered to these languages, with no publication deadline. The reference lists of the included studies and other relevant publications were also reviewed. Sources like Google Scholar were used to search for articles that matched eligibility criteria.

### 2.2. Elegibility Criteria

The format of the “PICOS” model [36] was used to select the studies for this systematic review. These criteria included the following: P (population): post-stroke patients; I (intervention): haptic feedback mechanisms; C (comparator): no treatment, other techniques/conventional therapy, no comparison; O (outcome): gait and/or balance; S (type of study): clinical trials (controlled and non-controlled, randomized and non-randomized), pilot studies, case series, and case reports. As specified in the PICO question, all study designs of primary research were included. Within these designs, studies involving the development of an intervention with haptic feedback were incorporated, as well as single-measure designs with haptic feedback focused on immediate effects. Thus, the research question of this review was as follows: Are haptic feedback mechanisms more effective than other techniques/conventional therapy in improving post-stroke balance and gait disorders?

The exclusion criteria included VR interventions and approaches with more than one type of feedback given contemporaneously. The VR case would not allow for an isolated study of haptic feedback as part of a larger system with other mechanisms, and the second situation would mix with effects from other types of feedback. Other kinds of studies such as letters to the editor, validation or design of a device studies, and conference proceedings were also excluded.

### 2.3. Assessment of the Methodological Quality and Risk of Bias

The methodological quality of the studies included in this review was evaluated using the Checklist for Measuring Study Quality (CMSQ), a tool well-suited for reviews that include both randomized and non-randomized trials [37]. It scores the following areas: reporting, external validity, internal validity, and statistical power. The level of evidence and strength of recommendation was determined using the classification system proposed by the Centre for Evidence-Based Medicine of Oxford (CEBM), which assigns different levels of evidence and grades of recommendation (A, B, C, and D) based on a study’s topic and design [38]. In addition, the PEDro scale [39] was used to assess the methodological quality of the RCTs included. This scale consists of 11 criteria, each awarding one point (except the first criterion) if met: (1) description of selection criteria; (2) random allocation; (3) concealed allocation; (4) similarity in groups at the beginning and end; (5) blinded subjects; (6) blinded therapists; (7) blinded evaluators; (8) follow-up of 85% of participants; (9) results reported for both intervention and control groups, including “intention to treat”; (10) statistical comparisons among groups; and (11) point and variability measurements [40]. Depending on the score on this scale, the articles were considered to be of excellent (9–10), good (6–8), fair (4–5), or poor (less than 4) methodological quality [41]. All the evaluations were carried out by two independent reviewers (M.G.-R. and E.M.). Any discrepancies were resolved by a third reviewer (C.L.-M.).

The Cochrane Risk of Bias Tool [42] was employed to categorize the risk of bias levels of studies included in the meta-analysis into three categories as follows: low, high, or unclear in the following areas: random sequence generation, allocation concealment, blinding of participants and personnel, blinding in outcome assessment, integrity of outcome data, selective reporting, and other potential sources of bias. Studies without a high risk of bias in any category were classified as high quality (1++), those with a high risk or two unclear risks as medium quality (1+), and others as low quality (1−). For the risk of bias assessment, the Cochrane Handbook for Systematic Reviews of Interventions [43] (RevMan^®^ Version 5.4) was used to evaluate articles, assigning ratings, performances, detections, attrition biases, and other potential biases. Statistical analysis and bias assessment were conducted using Review Manager software version 5.4^®^ (Cochrane Library, London, UK). Additionally, data were imported into the GradePro^®^ application (https://www.gradepro.org/, accessed on 8 July 2024) to evaluate the degree of recommendation into four levels (⨁◯◯◯ = Very Low; ⨁⨁◯◯ = Low; ⨁⨁⨁◯ = Moderate; ⨁⨁⨁⨁ = High) [44]. A sensitivity analysis was performed to evaluate the robustness of the results by sequentially excluding each study. *p*-values less than 0.05 were considered statistically significant.

### 2.4. Selection Process and Data Extraction

To select articles, a rigorous three-step procedure was implemented. The first step involved thoroughly searching the database, removing duplicates, and reading the titles and abstracts of the articles in order to identify suitable studies. In the second step, articles were excluded based on title or abstract, with further analysis conducted against predefined inclusion criteria. The third and final step entailed a comprehensive examination of the full text of the eligible articles to verify that the inclusion criteria were met. The entire process was developed by two independent authors (M.G.-R. and E.M.). The Rayyan research collaboration tool was used for the procedure. An additional reviewer (C.L.-M.) was consulted in case of disagreement.

Data were extracted from the selected studies and displayed in tables in a standardized manner, including author/s and year of publication, study design, sample characteristics (total number of participants, number of participants in each group, age, sex, time post-stroke, and type of stroke), details about interventions performed and haptic feedback procedures, dose specification of interventions (sessions/repetitions, the total number of sessions, follow-ups), variables, measuring tools, and results.

### 2.5. Data Synthesis and Statistical Analysis

Continuous variables were assessed using mean differences (MDs) along with 95% confidence intervals (CIs) and data were computed using random effects models because of the heterogeneity present in the included studies [45]. This heterogeneity in the studies was evaluated using the chi-square test and the I^2^ test, with a level of statistical significance based on a *p*-value < 0.05. The criteria to classify I^2^ values considered scores that ranged from 0% to 25%, indicating low heterogeneity; from 25% to 75%, indicating moderate heterogeneity; and more than 75%, indicating high heterogeneity [46].

The results of the meta-analysis were presented using a forest plot diagram and a funnel plot to assess potential publication bias among the studies. The asymmetry of the funnel plot was analyzed using the representation of the funnel plot and assessed with Egger’s test with a statistical significance level of 95% (*p*-value < 0.05). Data on static balance measured by CoP velocity were expressed in cm/s.

## 3. Results

**Figure 1:**
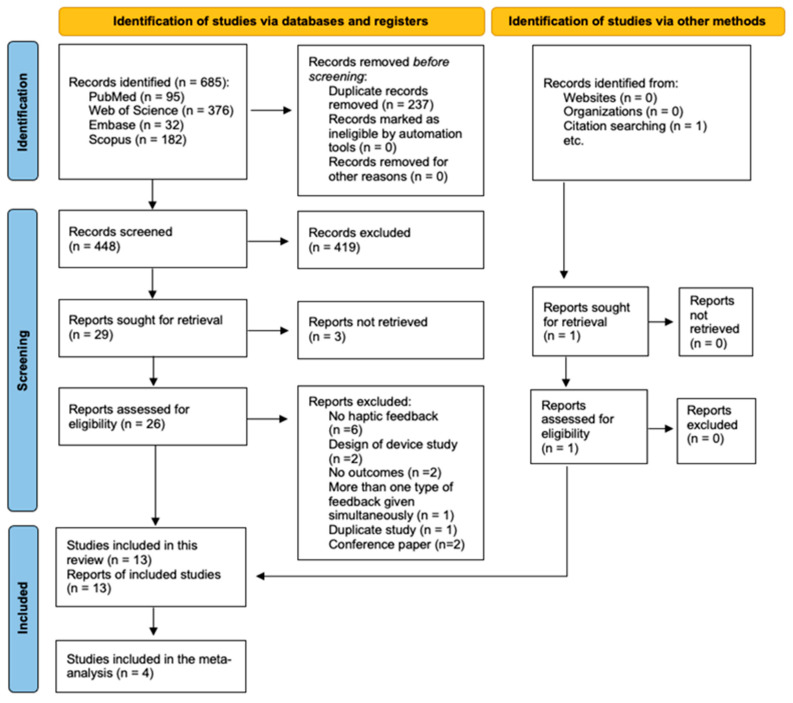
PRISMA 2020 flow diagram for new systematic reviews, which included searches of databases, registers, and other sources [35].

A total of 685 studies were identified. After the research procedures, 13 eligible trials met the inclusion criteria and were selected and included in this systematic review. A list with the records excluded and the reason for the exclusion is in the Supplementary Materials (Table S2). The reasons for excluding these reports are presented in the screening part of the flow diagram (Figure 1). A total of four studies were included in the meta-analysis [47,48,49,50].

### 3.1. Synthesis of Results

Specific information about the haptic feedback procedures is given in Table 2 The most relevant characteristics of studies included in this review are shown in Table 3.

### 3.2. Participant Characteristics

A total of 245 subjects (mean age: 60.40 years) were included in this review from the 13 selected studies. Regarding the time post-stroke, 58 subjects were in the early stages of recovery, while 187 subjects were in the chronic phase. All subject characteristics of each study are summarized in Table 4.

### 3.3. Intervention Characteristics

Related to the haptic feedbacks used in the studies, most were vibrotactile feedback [48,49,51,52,53,54,55]. Other studies used kinesthetic information [56,57], mixed haptic feedback (both vibrotactile and kinesthetic) [58], and electrical stimulation as haptic cues [47,50,59]. In six studies, feedback was given in the lower limbs [47,48,50,51,55,58], while in four studies, it was provided in the hips and trunk region [49,52,54]. Other regions such as the neck [56], arms [53], hands [57], and tongue [59] also received haptic information in the experimental procedures.

The different approaches to gait and balance in the pre–post-training studies had some characteristics in common. Balance training included both static and dynamic balance approaches. The interventions in two studies focused on weight shift (dynamic balance) [47,50]. Tasks such as bipedal stance and maintaining challenging postures were considered in four studies (static balance) [48,49,57,59], and a mixture of these two approaches was used in one study [54]. On the other hand, gait interventions were simpler and involved the action of walking under feedback conditions. Only one prospective study included gait as an outcome measure [54].

**Table 2:** Classification of haptic type and description of mechanism developed.

Authors (Year)	Haptic Feedback
Schonhaut et al. (2024) [52]	Vibration: Tractors attached to hip and trunk. Hip abductor vibration adjusted in real time according to pelvis movement. Trunk vibration as the other condition for comparison.
Lee et al. (2023) [47]	Electrical Stimulation: Low-frequency electrical output in LL triggered when weight shifting is detected by an insole pressure-measuring device.
Kim et al. (2022) [48]	Vibration: Pressure sensor-based vibrotactile biofeedback system that gives vibration inputs in calves related to torso tilt.
Lee et al. (2022) [56]	Kinesthetic: Tactile inputs to the neck, similar to a light touch in relation to the ML and AP directions.
Lee et al. (2021) [53]	Vibration: Haptic bracelet that gives feedback by vibration cues related to arm swing movement in gait.
Afzal et al. (2019) [55]	Vibration: Insoles with a Force-Sensitive Resistor in the foot to determine swing and stance phase and give vibrotactile stimuli accordingly in the swing phase of the paretic leg.
Yasuda et al. (2018) [54]	Vibration: Vibrotactile biofeedback bilaterally attached to the ASIS and PSIS enables perception of the center of pressure during balance tasks. The system gives the information to both the therapist and patient.
Afzal et al. (2018) [58]	Kinesthetic and vibration: Haptic cane device that provides kinesthetic information and vibrators that provide tactile feedback on the leg during the swing phase. Insoles to provide contact ground information are also part of the system.
Yasuda et al. (2017) [49]	Vibration: Vibrotactile biofeedback bilaterally attached to the ASIS and PSIS gave information about direction of body sway (CoP).
Ma et al. (2017) [51]	Vibration: Plantar force acquisition unit and a vibration feedback unit on the affected side of the patient. Vibrational cues given when excessive foot inversion occurred.
Kim. et al. (2015) [50]	Electrical Stimulation: FES therapeutic unit set to the minimum sensory stimulation level. Activated in LL when weight shift is achieved.
Afzal et al. (2015) [57]	Kinesthetic: Kinesthetic feedback given by Phantom Omni^®^ (patients’ hand grasping a handle). Feedback information in the form of light directional force indicating body movement to maintain balance.
Badke et al. (2011) [59]	Electrical Stimulation: Electrotactile feedback disposed in tongue (intraoral device that gives stimulus related to postural control).

AP: anteroposterior; ASIS: Anterior Superior Iliac Spine; FES: Functional Electrical Stimulation; LL: lower limb.

**Table 3:** Most relevant characteristics of the included studies.

Authors (Year)	Study Type (n)	Intervention and Dose	Variables: Outcome Measurement	Results
Schonhaut et al. (2024) [52]	CS (n = 40)	IED: Walking trials under different feedback conditions: no vibration, hip vibration and trunk vibration. Only one session (16 min/session).	Foot placement modulation: treadmill (other specifications not provided). Sacrum displacement and velocity (standing): method not specified.	Greater foot modulation in hip and trunk vibration modes (*p* < 0.01) and in constant mode of vibration (*p* = 0.01). Better standing and significant sacrum displacement (*p* < 0.01) with non-paretic side vibration. Paretic side vibration only affected to the sacrum displacement (*p* > 0.05).
Lee, K. (2023) [47]	RCT (n = 60) EG (n = 30) CG (n = 30)	EG: Balance training (BT) with WS as main exercise and electrical stimulation (ES) as feedback in LL. CG: Balance training without electrical stimulation. 30 sessions (50 min/session). 5 sessions/week. 6 weeks (total of 25 h). No follow up.	Static Balance Ability (sway speed and velocity moment): balance platform. Dynamic balance ability: TUG, FRT and BBS. Lower-extremity motor function: FM-LL. Activities of Daily Living: MBI.	Both groups showed improvement in all variables, but the experimental group showed greater improvement than the control (*p* < 0.05).
Kim et al. (2022) [48]	RCT (cross-over) (n = 24)	IED: Different feedback conditions while standing. All participants measured under three conditions in a randomized order: tactile BF (vibration); visual BF (mirror), and none feedback. 1 session for each condition (7.5 min/session; 3 sessions; 22.5 min). 24 h of washout between sessions.	Static Balance Ability (sway length and sway velocity): Wii Balance Board. Weight-Distribution Symmetry Index: Wii Balance Board.	Significant differences (*p* < 0.01) in sway length for tactile biofeedback. Tactile feedback also showed a significantly slower sway velocity and constant weight-distribution symmetry index compared with other conditions (*p* < 0.01).
Lee et al. (2022) [56]	CR (n = 1)	IED: Tasks of stance and gait balance protocol. Different conditions were carried out as Romberg and Straight-line tests with and without feedback. Only 1 session (min/session not specified).	Balance (trunk tilt): IMU sensor. Gait speed: IMU sensor.	Feedback device did not have effects on gait speed. No feedback condition and feedback conditions both showed improvement in balance.
Lee et al. (2021) [53]	CR (n = 1)	IED: Walking trials under different conditions: normal walk and different feedback in both paretic and non-paretic arms and backward and forwards movements. Only 1 session (min/session not specified).	Angle of arm swing: device on bracelets. Gait Parameters (velocity, stride length and SR): IMUs on lower limbs. ML and AP tilts: IMUs.	Arm swing modifications reached except in two feedback conditions (more complex feedback). Velocity and stride length increased in all feedback conditions. SR also improved under feedback conditions, as well as ML and AP tilts.
Afzal et al. (2019) [55]	CS (n = 8)	IED: Walking trials under different conditions: no feedback and feedback under different proportional or inversely proportional time and intensity changes. Only 1 session (min/session not specified).	Gait speed: handheld stopwatch. SR (calculated with ratio of stance-times): designed program connected to sensors and feedback device.	Statistically significant differences for SR in feedback trials. Significant differences between proportional time and intensity change feedback, and between inversely proportional time and intensity change feedback. No significant differences in gait speed.
Yasuda, K. et al. (2018) [54]	CS (n = 9)	Balance training (standing and WS) with vibrotactile BF. 8 sessions (45 min/session). 2 sessions/week. 4 weeks (total of 6 h). No follow up.	Patient’s postural stability (CoP pressure data in spatial variability, distance of sway and standard derivation of CoP time series): Wii Balance Board. Functional balance performance: BBS, FRT and TUG.	Significant improvement in CoP spatial variability, BBS, FRT and TUG between pre and post-tests (*p* > 0.05).
Afzal, MR. et al. (2018) [58]	CS (n = 10)	IED: Walking trials under different conditions: normal walk, tactile feedback, kinesthetic feedback at different walking speeds and both tactile and kinesthetic feedback at different walking speeds. Only 1 session (min/session not specified).	Stance Symmetry Ratio (SSR): insoles with sensors. Muscle activity: EMG. Balance (ML trunk tilt): smartphone.	In tactile, kinesthetic (normal speed) and tactile and kinesthetic (20% increase speed), SRR showed improvement. ML tilt was better in kinesthetic, and tactile and kinesthetic feedback conditions, but without statistical difference. Better muscle activity in kinesthetic feedback and tactile and kinesthetic feedback (normal speed).
Yasuda et al. (2017) [49]	CT (n = 17) EG (n = 9) CG (n = 8)	EG: balance task (bipedal stance) with BF information. 5 rep. of balance task (15 s each) with 1 min interval between rep. with BF. CG: balance tasks. 5 rep. of balance task (15 s each) with 1 min interval between rep. Only one session (5.25 min/session). No follow up.	Postural Stability (CoP spatial variability, CoP velocity of displacement and Mean CoP distance in the AP and ML directions): Wii Balance Board.	Only the CoP spatial variability and the mean distance in the ML direction were significantly lower in the experimental group (*p* > 0.05).
Ma et al. (2017) [51]	CS (n = 8)	IED: Walking trials under different conditions: biofeedback turned off (BFOff) and biofeedback turned on (BFOn). Only 1 session (min/session not specified).	Kinematic variables (foot, ankle, knee, hip and pelvic movements): Vicon Nexus 1.8.1 3D motion capture system. Plantar pressure distribution: in-shoe plantar pressure measurement system.	Stance (*p* < 0.05) and stride (*p* < 0.01) times significantly increased for both limbs. Foot inversion in swing phase of the affected limb significantly decreased in BFOn condition (*p* < 0.05). Peak knee flexion in swing phase and peak hip abduction in stance phase of the unaffected limb decrease (*p* < 0.05). In BFOn condition, plantar pressure distribution of affected limb increased significantly (*p* < 0.01) as well as average plantar pressure of both limbs (*p* < 0.05).
Kim. et al. (2015) [50]	RCT (n = 30) EG (n = 13) CG (n = 12)	EG: Weight shift (WS) training with electrical sensory stimulation feedback in LL (15 min/day) + CRehab (30 min/day). CG: General weight shift (WS) training (15 min/day) + CRehab (30 min/day). 20 sessions (45 min/session). 5 sessions/week. 4 weeks (total of 15 h). No follow up.	Balance in standing posture (CoP path lengths, CoP velocities and foot forces (FF)): Zebris Platform.	Improvements in CoP path length in experimental group with significant difference between groups (*p* < 0.05). Both groups showed improvement in FF, but there were better results in EG. Even though, no significant difference between groups for FF or CoP velocities.
Afzal et al. (2015) [57]	CS (n = 8)	IED: Balance trials while maintaining stance position under feedback and no feedback conditions. Only 1 session (min/session not reported).	Trunk tilt values: smartphone Body sway (mean velocity displacement, planar deviation, ML and AP trajectories): smartphone	Mean velocity displacement and planar deviation exhibited significant values when comparing no feedback and feedback conditions (*p* < 0.05).
Badke et al. (2011) [59]	CS (n = 29)	Segmental movement exercises and balance training (maintaining challenging postures) with TEF. 35 sessions (60 min/session). 2 sessions/day. 5 days/week. 8 weeks (total of 80 h). No follow up.	Balance: BBS. Gait ability: DGI. Balance and mobility: TUG, ABC. Quality of life: SIS.	Statistically significant improvement in BBS, DGI, TUG, ABC and almost all spheres of SIS.

Abbreviations: ABC: Activity-Specific Balance Confidence; AP: anteroposterior; ASIS: Anterior Superior Iliac Spine; BBS: Berg Balance Scale; BF: biofeedback; CG: control group; CR: case report; CRehab: Comprehensive Rehabilitation (Bobath or PNF); CS: case series; CT: clinical trial; CoP: center of pressure; DGI: Dynamic Gait Index; EG: experimental group; FM-LL: Fugl–Meyer Assessment Scale (lower limbs); FRT: Functional Reach Test; FU: follow-up; IED: Immediate effects design; IMU: Inertial Measurement Unit; LL: lower limb; MBI: Modified Barthel Index; ML: mediolateral; PNF: Proprioceptive Neuromuscular Facilitation; PSIS: Posterior Superior Iliac Spine; RCT: randomized controlled trial; reps: repetitions; SIS: Stroke Impact Scale; SR: Symmetry Ratio; SV: Swing-phase Vibration; TEF: tongue electrotactile feedback; TUG: Timed Up and Go Test; WS: weight shift.

**Table 4:** Description of the patient characteristics of each study.

Authors (Year)	Age (Mean)	Sex (M/F)	Phase and Time Post-Stroke (Mean)	Type of Stroke
Schonhaut et al. (2024) [52]	63.5	27/13	Chronic 69.5	Not reported
Lee, K. (2023) [47]	67.6 years	33/26	Chronic 15.25 ± 5.85 months	Thirty-seven ischemic Twenty-two hemorrhagic
Kim et al. (2022) [48]	63 years	18/6	Chronic 15.54 ± 9.00 months	Not reported
Lee et al. (2022) [56]	60 years	1/0	Early Subacute 37 days	Hemorrhagic
Lee et al. (2021) [53]	64 years	1/0	Early Subacute 26 days	Ischemic
Afzal et al. (2019) [55]	54.5 years	6/2	Early Subacute 23.9 ± 9.3 days	Five ischemic Three hemorrhagic
Yasuda et al. (2018) [54]	65.8 years	7/2	Chronic 81.56 months	Four ischemic Five hemorrhagic
Afzal et al. (2018) [58]	57.7 years	6/4	Early Subacute 62.5 ± 26.6 days	Five ischemic Five hemorrhagic
Yasuda et al. (2017) [49]	65.1 years	13/4	Chronic 38.16 months	Ten ischemic Seven hemorrhagic
Ma et al. (2017) [51]	53.5 years	8/1	Chronic 45 months	Six ischemic Two hemorrhagic
Kim. et al. (2015) [50]	59.6 years	17/8	Late Subacute 12.8 ± 7.6 weeks	Not reported
Afzal et al. (2015) [57]	52 years	6/2	Late Subacute 70.0 ± 41.4 days	One ischemic Seven hemorrhagic
Badke et al. (2011) [59]	59 years	20/9	Chronic 52.2 ± 34.8 months	Not reported

### 3.4. Methodological Quality and Risk of Bias

The included studies were heterogeneous, including three RCTs [47,48,50], one CT [49], seven CSs [51,52,54,55,57,58,59], and two CRs [53,56]. According to CEBM Levels of Evidence, only the first three presented a good grade of recommendation and level of evidence (A, 1b). Regarding the CMSQ, ten studies scored low quality (less than 50%), two scored medium quality (between 50 and 65%), and one scored high quality with more than 65%. The complete CMSQ scores are presented in the Supplementary Material of this article (Table S3). The RCTs’ methodological qualities were also measured with the PEDro scale, resulting in poor [50] and good [47,48] methodological qualities after evaluation (Table 5).

**Figure 2:**
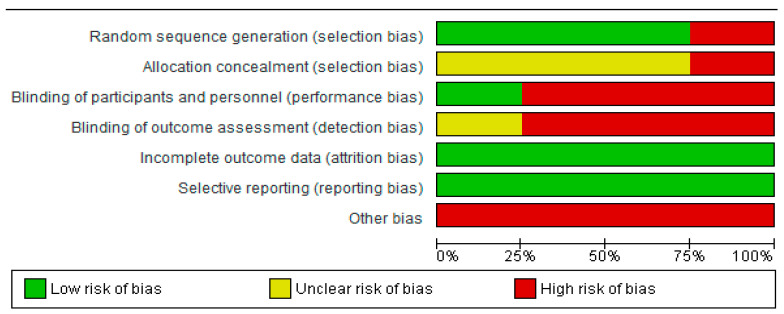
Bias assessment plot of all included studies.

**Figure 3:**
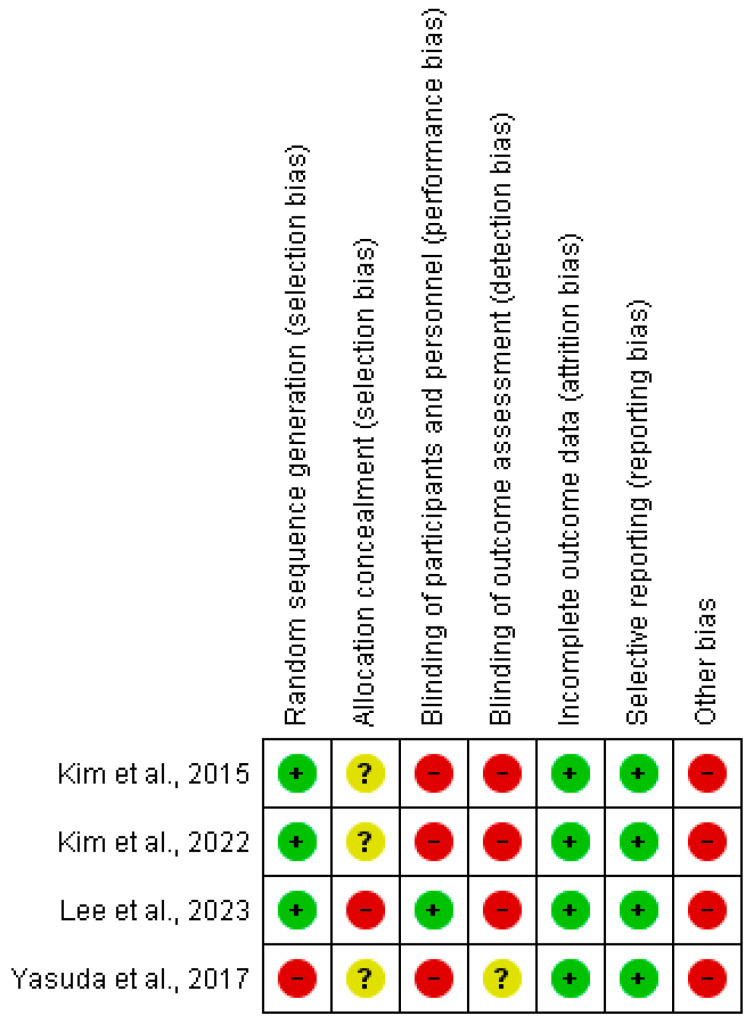
Bias assessment shown as a one-to-one summary plot [47,48,49,50]. Red= high risk; Green= low risk; Yellow/?: unclear risk; +/− = risk percentage.

The risk of bias of the studies included in the meta-analysis was assessed using RevMan 5.4©, and it is represented in Figure 2 and Figure 3 by bias assessment plots of the studies included and by a one-to-one-summary plot. Randomization was evident in 75% of the included studies; allocation concealment was doubtful in around 75% of the included studies; with approximately 25% blinding of participants and personnel; and 25% were doubtful for the blinding of outcome evaluation.

The degrees of recommendation observed for the outcome assessed in the meta-analysis showed a very low degree of recommendation according to GRADE PRO^©^ (Table 6).

**Figure 4:**
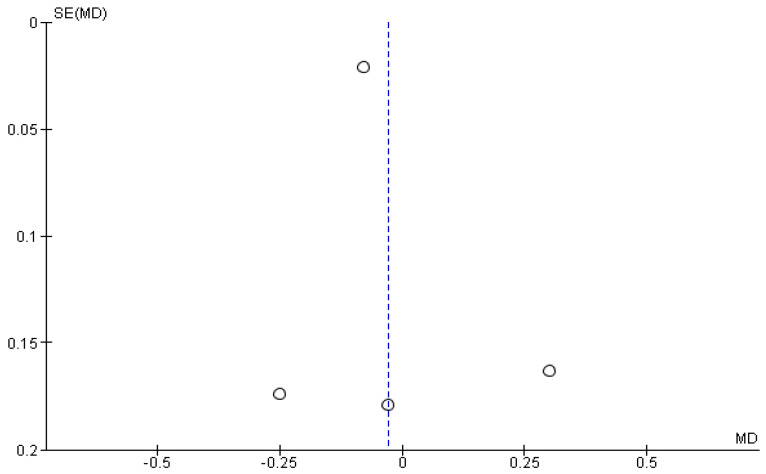
Analysis of publication bias by a funnel plot.

For publication bias, the funnel plot assessed shows a symmetric funnel plot, with the strongest studies concentrated in the center (Figure 4). However, the number of included studies is very small, and the graph should be interpreted with caution.

### 3.5. Results of the Meta-Analysis

In four clinical trials involving 149 patients, with 75 in the intervention group and 74 in the control group, the efficacy of balance training with haptic feedback on the improvement in CoP velocity as opposed to balance training without haptic feedback information in patients with stroke was compared [47,48,49,50].

**Figure 5:**
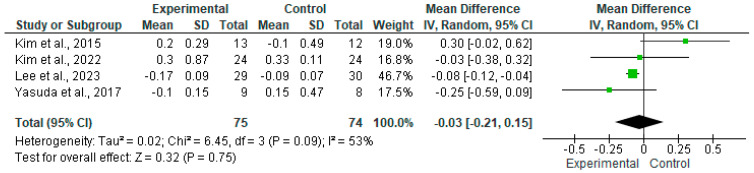
Efficacy of haptic feedback interventions in the velocity of CoP [47,48,49,50].

A higher average speed was observed in the experimental group compared with the control group. No statistically significant differences were found between the two groups (*p* = 0.75), with an MD of −0.03 and a 95% confidence interval of −0.21 to 0.15. Significant heterogeneity among the studies was shown (I^2^ = 53%, *p* = 0.09) (Figure 5). This seems to indicate that the intervention group in three of the studies included in the meta-analysis showed better results compared with the control group [47,48,49]. In contrast, in the study by Kim et al. (2015) [50], a higher velocity in individuals with stroke was shown when treated with a control intervention.

### 3.6. Immediate Effects on Balance and Gait

Eight studies [48,51,52,53,55,56,57,58] investigated the immediate effects of haptic feedback mechanisms in the main variables of this review by carrying out single-measure (or simultaneously measured) designs. On the one hand, related to balance, three studies assessed immediate feedback effects on this variable. One measured it with CoP information (displacement and velocity) [48], another with trunk tilt values [56], and the third with both measurements [57]. There were statistically significant differences in CoP information under feedback conditions in the first study [48], the second study showed improvement in trunk tilt under both feedback and no feedback conditions [56], and the third showed significant differences between feedback and no feedback conditions in the mean displacement and planar deviation of body sway [57]. Other balance-related measures such as the weight distribution index, assessed in the study by Kim et al. [48], also showed statistical differences under feedback conditions.

On the other hand, six studies reported haptic feedback immediate effects in gait [51,52,53,55,56,58]. Those effects were measured by insoles [51,58], Inertial Measurement Units (IMUs) [53,56], other procedures such as 3D analysis (Vicon Motion System) [51], or manual methods (stopwatch) [55]; one study did not report the outcome measurement [52]. The most measured variables were gait speed [53,55,56] and the Symmetry Ratio (SR) [53,55,58]. Gait speed under feedback conditions improved in one study [53], while SR improved in all the studies that measured it.

### 3.7. Post-Training Effects on Balance

#### 3.7.1. Static Balance

Four studies showed post-training results for static balance [47,49,50,54] given CoP information. All studies used stabilometric platforms to assess this variable. Two out of the four studies [47,50] showed significant results for all measured variables related to CoP under feedback conditions. In the case of Yasuda et al.’s studies [49,54], the results were more modest, being statistically different under feedback conditions from the CoP spatial variability in both studies and the displacement in the mediolateral (ML) direction in the second study.

Regarding the results from the meta-analysis carried out for this variable, a higher average speed in CoP velocity in experimental groups of the studies included in the analysis was found. Even though no statistically significant differences were found between the two groups, significant heterogeneity among the studies was shown (I^2^ = 53%, *p* = 0.09).

#### 3.7.2. Dynamic Balance

Three studies reported information about this variable [47,54,59] and measured it through specific tests such as the Berg Balance Scale (BBS), the Timed Up and Go Test (TUG) [47,54,59], and the Functional Reach Test (FRT) [47,54]. All these studies showed positive results. Lee et al.’s [47] study reported significant results for the experimental group compared with the control group (*p* < 0.05). The studies carried out by Yasuda et al. [54] and Badke et al. [59] found significant post-training improvement in the intervention group (*p* < 0.05).

### 3.8. Post-Training Effects on Gait

Only one study investigated the haptic feedback post-training effects on gait [59]. In that study, gait performance was assessed using the Dynamic Gait Index (DGI). The results of that study [59] showed statistical improvement after intervention (*p* < 0.001).

### 3.9. Other Functional Outcomes and Post-Training Effects

Lower extremity motor function was assessed in one study [47], showing significantly greater improvement in the experimental group (*p* < 0.05). Other variables related to daily living were measured, such as the Modified Barthel Index [47], with significantly better results for the experimental group or the Stroke Impact Scale (SIS) [59] that showed significant improvement in all spheres except for SIS Strength, SIS Memory, SIS Communication, and SIS Emotions.

## 4. Discussion

This review analyzed studies investigating the effects of adding haptic feedback in balance and gait disorders approaches. The meta-analysis showed that there were no statistically significant differences between the two groups. However, a higher average speed was observed in the experimental group compared with the control group in three studies. Only in the study by Kim et al. [50] did the control group show better results than the experimental group. Possible causes for these results are discussed in the following subsections. All studies included in this meta-analysis focused on the measurement of static balance (CoP velocity). Three employed a prospective design [47,49,50], while one used a cross-sectional setup [48]. It is important to note that even though it was considered a pre-post intervention, the study developed by Yasuda et al. [49] was practically an intervention with immediate effects because of the short duration of the session. However, it was not categorized as such because of the design of pre- and post-intervention measures, as opposed to single-measure designs.

Regarding the general information derived from this review, several interesting observations can be made. More than half of the studies included focused on stroke patients in the chronic phase (seven out of thirteen). In interventions carried out in a more chronic stage, recovery might be more attributable to the intervention than to spontaneous recovery (which predominates in acute stages) [60]. However, some research emphasizes the importance of early treatment in stroke cases, with most experts recommending starting within 48 h to avoid poorer outcomes [61]. Despite the potential confounding effects from natural recovery, it is crucial to design effective and intensive interventions for acute patients [62]. Feedback strategies, with their adaptive and intuitive features, could provide significant research opportunities in these early stages [53].

In relation to different haptic feedback mechanisms developed in the settings of the studies included in this review, it is important to highlight the predominant use of vibrotactile feedback. Evidence available in the field indicates that vibrotactile feedback-based intervention may serve as a safe and effective complementary sensory–motor approach for balance and gait rehabilitation in patients with neurological and cerebrovascular conditions [11]. This aligns with the results of this review, as all studies that used vibrotactile feedback achieved positive outcomes for the experimental or intervention group. However, in this review, a heterogeneous set of feedback-based interventions was provided, which also included approaches with electrical and kinesthetic stimulation, and these also yielded good results.

Finally, another general aspect to take into consideration is not only the statistical significance but also the clinical significance of the results provided by the studies in the present review. The concept of minimal clinically important difference (MCID) represents a threshold value that signifies a change perceived and detected by the patient following an intervention, differing from a statistically significant difference [63]. It is crucial to correlate statistically significant results with their clinical impact to avoid misinterpreting study findings, which could lead to unnecessary patient exposure to therapies [64]. Additionally, the Minimal Detectable Change (MDC) is a psychometric property of interest, being the smallest detectable change that exceeds the measurement error, with a specified level of confidence [63]. Only two studies included in this review reported information about this aspect [54,59].

### 4.1. Immediate Effects on Balance

Some differences in the interventions and results among the three studies that addressed the immediate effects of balance should be highlighted. First, Kim et al. [48] and Afzal et al. [57] established standing as the position to be maintained during the feedback application. Lee et al. [56] established a design where two tasks were developed for measuring the variables. These tasks were the Romberg test and tandem walking, and balance was assessed based on trunk movement during these tasks. The development of these demanding tasks contrasts with the simplicity of the measurement in the studies by Kim et al. [48] and Afzal et al. [57]. It appears that one possible explanation is that the action of haptic feedback during the performance of a more complex task might not reach its full potential, compared with simpler tasks, in cases where immediate effects are pursued. However, feedback during complex task execution over long intervention periods could yield equally positive results, as evidenced by other studies included in this review [47,49,50,54,59].

### 4.2. Immediate Effects on Gait

In terms of immediate effects on gait, the good results for the Symmetry Ratio (SR) under varying speeds are especially interesting, with a 20% increase reported in Afzal et al.’s study [58]. Evidence highlights that the most efficient gait pattern is symmetrical, as gait asymmetry is linked to increased energy expenditure [65]. In the chronic stage post-stroke, 55.5% of individuals display temporal asymmetry [55]. Typically, walking training on a treadmill is widely used to assess asymmetry issues, although some authors emphasize that this approach should be complemented with overground walking [3]. The reason is that treadmill speeds do not always translate to overground walking speeds [3]. The design proposed by Afzal et al. [58] aligns with the evidence, as the feedback device supports overground walking while allowing for increased speed. SR also showed statistically significant differences under feedback conditions in the other studies that measured it [53,55]. Approaches like those implemented in the mentioned studies could be an excellent option for future prospective designs where long-term changes in this important variable are observed. Kinematic variables such as stride length also improved under the feedback approach [51,53], as well as stance time, stride time, foot inversion and plantar pressure [51], and the angle of arm swing [53].

Finally, gait speed was assessed as an outcome measure in three studies [53,55,56], and it improved under haptic feedback conditions in one of them [53]. The other studies [55,56] did not show significant differences in speed under feedback conditions compared with control conditions. One possible explanation could be the type of haptic feedback used in these studies. In the study with positive results, the feedback device was worn on the arms, whereas in the others, the devices were attached to the neck or shoes as insoles. It may be interesting to further investigate the effects of haptic feedback on arm swing and its influence on variables such as gait speed. This would be in line with the most recent evidence that focuses on studying this kinematic aspect and highlights its importance in post-stroke gait recovery [66].

### 4.3. Post-Training Effects on Balance

#### 4.3.1. Static Balance

Studies measuring static balance after an intervention period [47,49,50,54] focused on CoP measurement. Prior studies highlighted the excellent test–retest reliability of laboratory-based force platforms [67] and the accuracy of measuring balance control using spatiotemporal variables such as CoP [68]. Two of the studies examining the post-training effects of haptic feedback on balance reported statistically significant results in all CoP variables measured [47,50], but in the studies of Yasuda et al. [49,54], the results showed improvement in only one of all the CoP variables assessed (spatial variability and ML direction displacement respectively). Regarding this last-mentioned variable of ML displacement, a recent paradigm shift proposed by some authors [69] is intriguing as they advocate for considering the relationship between greater ML sway and lower fall risk. In contrast to other evidence [70], it could be an interesting hypothesis to consider that stroke survivors with a greater ML sway range and, hence, more asymmetry may prefer a natural asymmetry while standing that allows them to walk without compromising their balance and thus avoiding falls [69]. The results from the studies included in this review could be conceived through this more functional prism, potentially leading to different conclusions. However, Nardone et al. [65] established that CoP-related asymmetry affected gait development by increasing the time and effort needed to transfer weight to the affected limb.

Regarding the meta-analysis results, the study by Kim et al. [50] showed better results for the control group in the velocity of CoP after intervention. The approach developed in their study included electrical sensory stimulation feedback in lower limbs related to the performance of weight-shifting tasks (experimental group) and general weight-shift intervention (control group). The results of their study can be specifically compared with the study conducted by Lee et al. [47] as both used the same type of haptic feedback in the interventions (electric). However, it is interesting to note an aspect that may have influenced the mentioned results. While in the study conducted by Lee et al. [47], the intervention of the experimental group lasted 50 min per session with haptic feedback for the entire duration, the experimental group in the study by Kim et al. [50] received haptic feedback for 15 min of the session, completing the remaining time with 30 min of conventional therapy. Thus, although both studies ultimately had nearly the same total duration of therapy per session (approximately 50 min) and used the same type of haptic feedback, they did not have the same amount of time dedicated to the feedback mechanism under study. This may have influenced the reported results.

#### 4.3.2. Dynamic Balance

In dynamic balance, BBS, TUG, and FRT were used. When analyzing the results, it is important to consider the psychometric properties of these clinical tests. The MDC value given by the authors that assessed BBS was from 2.5 to 4.6 [54,59], while another study in the literature cites a value of 1.43 points [71]. Regarding those scores, 72.4% of patients reported in Badke et al.’s study [59] reached a real change in BBS. In the case of Yasuda et al.’s study [54], patients would have a detectable change if they had taken other difference values [71], in contrast to what was reported by the authors. Lee et al. [47] did not provide psychometric values for BBS; however, based on the literature, only patients in the experimental group would have reached the threshold.

For the TUG test, the studies by Yasuda et al. [54] and Badke et al. [59] reported psychometric values based on the Flansbjer et al. study [72]. However, that study provided the Smallest Real Difference (SRD), a concept expressed as a percentage independent of the unit of measurement. That study established this SRD% for the TUG at 23% to indicate a real clinical change. The authors argued that these psychometric measures better fit clinical reality compared with when results are constrained to static value thresholds [72].

Finally, regarding the FRT, patients in both studies who reported data for this test [47,54] had good results for experimental conditions. Notably, both interventions used the weight shift-based approach. Only the study developed by Yasuda et al. [54] reported psychometric data and crossed the threshold reported by the authors.

### 4.4. Post-Training Effects on Gait

The unique study measuring this variable after a treatment period [59] made it difficult to draw conclusions. In addition, even though that study measured gait as an outcome, the intervention did not include specific gait approaches. Nevertheless, improvements in other variables such as balance could be interesting, as it has been previously stated that good balance is important for stepping patterns, gait velocity, and the initial stages of gait training in individuals recovering from stroke [6]. Badke et al. [59] measured gait improvement through the DGI scale, reported the psychometric characteristics of this scale, and analyzed results accordingly. The DGI is scored on a four-point scale from 0 to 3, with 0 indicating severe impairment and 3 representing normal walking ability, with the highest possible total score of 24 [73]. Information regarding the psychometric properties of DGI is limited, but Badke et al. [59] established its MDC at 2.7 points based on previous studies [73]. Related to that, almost half of the participants (44.8%) in the study reviewed would have surpassed this threshold [59].

An important consideration is that current evidence supports the use of instrumented gait analysis over traditional observational methods. Traditional methods rely on the expertise of the observer, whereas instrumented systems provide precise information [74]. In this review, all studies investigating the immediate effect of interventions on gait employed instrumented systems [51,52,53,55,56,58], whereas the study assessing this variable post-intervention did not. One possible explanation could be that in methodological designs where measurements are only taken on a single day, it is easier to access resources of this kind compared with designs that require pre/post-tests. However, the use of these resources should be strongly recommended in those studies.

### 4.5. Other Outcomes

The significantly greater improvement in the experimental group in the Lee et al. study [47] also reached the MCID for the Fugl–Meyer of lower limb scale (established by evidence at six points) [75]. Other variables related to daily living, such as the Modified Barthel Index, were measured [47]. Although the authors did not report values for it, evidence has established the MCID for the Barthel Index from 4 to 5 points [76]. According to that, the mean values of the experimental group of that intervention surpassed the MCID, while the control group did not. These results align with the statistical results shown in another study [47], providing additional information about the real change experienced after the approach was developed. Finally, the MCID for SIS has been previously established as 15 points [77]. According to that, results from the study developed by Badke et al. [59], which reported MCID values, showed that the domain of the scale where most patients reached this threshold was SIS mobility. However, less than half of the sample achieved this significant difference.

### 4.6. Clinical Implications

Weight shift training with haptic feedback for balance recovery of post-stroke patients was present in some prospective studies included in the present review [47,50,54]. The outcome measures were mainly scales, such as BBS, but most of them used the assessment of CoP. According to current evidence, many authors have focused on these weight-shifting interventions to improve balance recovery after a stroke. For example, Ostrowska et al. [78] reached good CoP variable improvements after SPIDER therapy focused on restoring balance through weight shift in these patients. This aligns with what other authors have investigated, such as Park et al. [79], who stated the importance of lateral weight transfer as a key element in the physiotherapeutic approach after a stroke. Also, some studies have pointed out this aspect, relating weight shift improvement with gait recovery [80,81]. All this information is consistent with the results presented in our study, despite limitations due to methodological restrictions of some of the articles included in the present review. Haptic feedback therapy could be aligned with the aforementioned interventions, providing good results in balance recovery (specifically in CoP measures).

Similar to the work developed by Ostrowska et al. [78], haptic feedback has a fundamental characteristic that sets it apart from other procedures: it serves as a valuable aid to the therapist. As some previous studies have indicated, it is sometimes challenging for the therapist to stay vigilant to all necessary corrections when treating post-stroke patients with body adjustment problems [82]. Therefore, haptic feedback could effectively address this need by providing information typically given by the therapist, allowing the therapist to focus on other corrections. Even a study included in this review provides a type of “empathetic haptic feedback” that delivers haptic corrections related to patient performance both to the patient and to the physiotherapist [54]. This significantly expands the range of treatment possibilities and proposes initiatives of great interest for future RCTs.

Another aspect derived from the discussion of the present study is the need to implement a functional approach in assessments, considering both clinical importance and statistical significance. Although this point was previously addressed in this manuscript, the development of this aspect is essential for adding greater significance to clinical interventions. Alongside this, in accordance with the principles of the International Classification of Functioning (ICF), it is important to consider both the individual and their biopsychosocial context, embracing a patient-centered approach [83]. However, only two studies in this review incorporated this perspective [47,59].

Finally, haptic feedback interventions seem to be secure and have good acceptance by patients. Some experiences of subjects such as confidence with the feedback-providing device [56] or comfort in its use [51,56] support this fact. Nevertheless, no study reported data on adverse effects.

### 4.7. Study Limitations and Future Research Lines

The limitations of this review are primarily attributed to the heterogeneity and the small sample observed across the included studies in terms of methodological designs. Similarly, another limiting aspect is the low methodological quality shown in the articles included in the present review, as well as the risk of bias displayed. A methodological limitation is that some reports that were not retrieved could not be found because of the absence of contact with their authors. In addition, it is important to acknowledge that certain search terms related to stroke (e.g., “brain ischemia” or “brain hemorrhage”) were not included in the search strategy. This limitation could have contributed to the omission of some studies, potentially impacting the review results. This aspect will be taken into consideration in future research.

In terms of results, only one study conducted a post-training gait assessment, while most measures of this variable were conducted in studies focusing on immediate effects. In those, some good results, such as those observed in SR, could be related to spontaneous recovery due to the acute phase intervention in all studies that report results for this variable. The results from these studies arise from immediate effects that occur simultaneously with the application of haptic feedback. Therefore, there is insufficient information to draw robust long-term conclusions.

Moreover, there was a noticeable lack of comparative analysis of the psychometric properties of outcomes, as only two studies incorporated such assessments. In addition, none of the interventions reported follow-up evaluations to assess the sustainability of the observed effects over time. Finally, the meta-analysis included only a few articles, and the results must be interpreted with caution because of this limitation. No statistically significant differences were found between the two groups, so the greater effectiveness of the experimental intervention over the control intervention cannot be supported. In this regard, it could be interesting to consider other therapies that might be used alongside haptic feedback as an intervention. Given the poor results derived from the meta-analysis, combining therapies could be a promising strategy and a significant prospect for future research.

These factors underscore the need for more comprehensive and standardized methodologies in future research studies within this field. Larger sample sizes and follow-up periods are also necessary.

## 5. Conclusions

Haptic feedback mechanisms appear to have good effects in balance recovery, especially in CoP speed improvement, although results should be considered with caution because of the limitations of this review. In addition, the meta-analysis did not find significant differences between the haptic feedback approach and the control interventions developed in the included studies. Weight shift training with haptic feedback mechanisms seems to be a commonly implemented procedure for balance recovery, consistent with the current evidence. Longer intervention periods are necessary to assess the development of gait using these mechanisms, while single-measure designs showed positive results of immediate effects on gait variables such as SR. The vibration-based haptic feedback mechanism appears to be one of the most commonly used and reports good results, although other approaches, such as those using kinesthetic or electrical feedback have also revealed promising results. Further research is necessary in order to determine the effectiveness of haptic feedback mechanisms, including studies with larger sample sizes, follow-up periods, and more robust methodologies.

## Figures and Tables

**Table 1:** Search strategies for different databases.

Databases	Search Strategy
Embase	“feedback system” AND (“tactile stimulation” OR “vibration sense” OR electrostimulation) AND “cerebrovascular accident” AND (training OR program OR exercise OR intervention OR rehabilitation OR physiotherapy OR therapy) AND (gait OR balance OR “lower limb” OR walking OR mobilization).
Medline/PubMed Web of Science Scopus	Feedback AND (haptic* OR vibr* OR electric* OR tactile) AND stroke AND (training OR program* OR exercise OR intervention OR rehab* OR physiotherap* OR therapy) AND (gait OR balance OR “lower limb” OR walk* OR ambul*).

**Table 5:** PEDro scale for the methodological quality assessment of randomized controlled trials [39].

Authors (Year)	Total	Items										
		1	2	3	4	5	6	7	8	9	10	11
Lee et al. (2023) [47]	8/10	Yes	1	1	1	0	0	1	1	0	1	1
Kim et al. (2022) [48]	6/10	Yes	1	0	1	0	0	0	1	1	1	1
Kim et al. (2015) [50]	4/10	yes	1	0	1	0	0	0	0	0	1	1

**Table 6:** Degrees of recommendation.

Certainty Assessment							№ of Patients		Effect		Certainty	Outcome
№ of Studies	Study Design	Risk of Bias	Inconsistency	Indirect Evidence	Imprecision	Other Considerations	EG	CG	Relative (95% CI)	Absolute (95% CI)		
4	CTs	Very serious	Not serious	Serious	Not serious	Publication bias is strongly suspected Low association	75	74	-	MD −0.03 (−0.21 to 0.15)	⨁◯◯◯ Very Low	CoP velocity

Abbreviations: EG: experimental group; CG: control group; CI: confidence interval; MD: mean difference; CTs: clinical trials. ⨁◯◯◯ = level of recommendation.

## Data Availability

No new data were created or analyzed in this study. Data sharing is not applicable to this article.

## Supplementary Materials

The following supporting information can be downloaded at https://www.mdpi.com/article/10.3390/jpm14090974/s1, Table S1: PRISMA checklist; Table S2: Records excluded and reasons; Table S3: CMSQ scores.
