# Supplementary material for: Effects of Haptic Feedback Interventions in Post-Stroke Gait and Balance Disorders: A Systematic Review and Meta-Analysis
Source: J Pers Med. 2024 Sep 14;14(9):974. doi: 10.3390/jpm14090974 (PMC11433178; doi:10.3390/jpm14090974)
Supplement: Supplementary file 1 [file jpm-14-00974-s001.zip › Table S3.pdf]

SUPPLEMENTARY MATERIAL TABLE S3. CMSQ scores.

	Studies							
	Schonhaut et al. (2024)	Lee et al. (2023)	Kim et al. (2022)	Lee et al (2022)	Lee et al (2021)	Afzal et al. (2019)	Yasuda et al. (2018)	Afzal et al. (2018)
**Checklist items for Measuring Quality**								
1. Study Quality								
Hypothesis / aim	1	1	1	1	1	1	1	1
Outcomes	1	1	1	1	1	1	1	1
Patients' characteristics and eligibility criteria	1	1	1	1	1	1	1	1
Interventions	1	1	1	1	1	1	1	1
Confounders	0	0	0	0	0	0	0	0
Findings	1	1	1	1	1	1	1	1
Random Variability	1	1	1	1	1	1	1	1
Adverse Events	0	0	0	0	0	0	0	0
Lost to Follow Up	0	1	0	0	0	0	0	0
Probability Values	1	1	1	1	1	1	1	1
2. External Validity								
Source Population	0	1	1	0	0	0	0	0
Illustrative sample	0	1	1	0	0	0	0	0
Illustrative treat	1	1	1	1	1	1	1	1
3. Internal Validity (Study Bias)								
Blinding of subjects	0	0	0	0	0	0	0	0
Blinding therapists assessing results	0	1	0	0	0	0	0	0
"Data dredging"	1	1	1	1	1	1	1	1
Follow-up Adjusts	0	0	0	0	0	0	0	0
Statistical Tests	1	1	1	1	1	1	1	1
Compliance	1	1	1	1	1	1	1	1
Accurate outcome measures	1	1	1	1	1	1	1	1
4. Internal Validity (confounding and selection bias)								
Same recruitment source of groups	0	1	1	0	0	0	1	0
Same recruitment period	0	1	1	1	1	0	0	1
Randomization	0	1	1	0	0	0	0	0
Concealment	0	1	1	0	0	0	0	0
Adjustment for confounding in the analysis	0	0	0	0	0	0	0	0
Lost to follow-up	0	1	0	0	0	0	0	0
5. Power								
Effect	0	1	0	0	0	0	0	0
**Total score (percentage %)**	37.5%	68.75%	56.25%	40.62%	40.62%	37.5%	40.62%	40.62%

SUPPLEMENTARY MATERIAL TABLE S3. CMSQ scores *(continue).*

	Studies				
	Yasuda et al. (2017)	Ma et al. (2017)	Kim et al. (2015)	Afzal et al. (2015)	Badke et al. (2011).
**Checklist items for Measuring Quality**					
6. Study Quality					
Hypothesis / aim	1	1	1	1	1
Outcomes	1	1	1	1	1
Patients' characteristics and eligibility criteria	1	1	1	1	1
Interventions	1	1	1	1	1
Confounders	0	0	0	0	0
Findings	1	1	1	1	1
Random Variability	1	1	1	1	1
Adverse Events	0	0	0	0	0
Lost to Follow Up	0	0	1	0	0
Probability Values	0	1	1	0	1
7. External Validity					
Source Population	0	0	1	0	0
Illustrative sample	0	0	1	0	0
Illustrative treat	1	1	1	1	1
8. Internal Validity (Study Bias)					
Blinding of subjects	0	1	0	0	0
Blinding therapists assessing results	0	0	0	0	0
"Data dredging"	1	1	1	1	1
Follow-up Adjusts	0	0	0	0	0
Statistical Tests	1	1	1	0	1
Compliance	1	1	1	1	1
Accurate outcome measures	1	1	1	1	1
9. Internal Validity (confounding and selection bias)					
Same recruitment source of groups	1	0	1	1	0
Same recruitment period	1	0	1	1	0
Randomization	0	0	1	0	0
Concealment	0	0	0	0	0
Adjustment for confounding in the analysis	0	0	0	0	0
Lost to follow-up	0	0	0	0	0
10. Power					
Effect	0	0	0	0	0
**Total score (percentage %)**	40.62%	40.62%	56.25%	37.5%	37.5%
